# A Comprehensive Review of Arachnoid Cysts

**DOI:** 10.7759/cureus.83894

**Published:** 2025-05-11

**Authors:** Jen-Yeu Wang, Hamid Hadi, Mohammad Arshad, Eric Whitney

**Affiliations:** 1 Neurosurgery, California University of Science and Medicine, Colton, USA; 2 Neurosurgery, Desert Regional Medical Center, Palm Springs, USA; 3 Neurosurgery, Arrowhead Regional Medical Center, Colton, USA

**Keywords:** arachnoid cyst, cystoperitoneal shunting, endoscopic cyst fenestration, management of arachnoid cyst, microsurgical cyst excision

## Abstract

Arachnoid cysts are cerebrospinal fluid (CSF)-filled sacs that develop within the arachnoid membrane surrounding the brain or spinal cord, often remaining asymptomatic but occasionally causing neurological symptoms due to mass effect or CSF flow obstruction. The optimal management of these cysts remains debated, balancing conservative observation with surgical intervention. This review analyzes the epidemiology, pathophysiology, clinical presentation, diagnostic strategies, treatment options, and future management directions for arachnoid cysts. With a prevalence of approximately 1-2% and a male predominance, these cysts most commonly occur in the Sylvian fissure and posterior fossa. Magnetic resonance imaging (MRI) serves as the gold standard for diagnosis, with diffusion-weighted imaging and cine phase-contrast MRI playing critical roles in differentiating communicating versus non-communicating cyst types and assessing CSF dynamics. Asymptomatic cysts are typically managed conservatively with periodic neuroimaging follow-up, while surgical intervention is reserved for symptomatic cases, particularly those associated with hydrocephalus, seizures, or focal neurological deficits. Treatment strategies include endoscopic fenestration, microsurgical fenestration, and cyst-peritoneal shunting, with endoscopy offering a less invasive option but carrying a higher recurrence risk. Advances in neuroimaging, surgical techniques, and artificial intelligence-driven predictive modeling are refining treatment approaches, while emerging research into molecular mechanisms and minimally invasive robotic-assisted surgery may further optimize outcomes. Future developments in personalized, risk-stratified management protocols may reduce the need for invasive interventions and improve long-term prognoses.

## Introduction and background

Arachnoid cysts are cerebrospinal fluid (CSF)-filled sacs that develop within the arachnoid membrane surrounding the brain or spinal cord. They are considered benign, non-neoplastic lesions that can be either congenital or acquired, with congenital cases accounting for the majority of diagnosed cysts [1]. These cysts are often incidental findings on neuroimaging studies, with many remaining asymptomatic throughout a patient’s lifetime. However, in cases where cysts enlarge or exert mass effect on adjacent neural structures, they can lead to neurological symptoms requiring medical or surgical intervention [2].

The first descriptions of arachnoid cysts date back to the early anatomical studies of the brain, but their clinical significance has been recognized only in the past few decades with the advent of advanced neuroimaging techniques. Modern magnetic resonance imaging (MRI) has facilitated the identification and characterization of these cysts, leading to increased awareness and more precise clinical management [3].

The importance of studying arachnoid cysts lies in their variable clinical presentation and the ongoing debate over the best management strategies. While conservative monitoring is sufficient for most cases, surgical intervention may be warranted for symptomatic cysts causing hydrocephalus, seizures, or focal neurological deficits [4]. Additionally, emerging research is uncovering potential genetic and molecular mechanisms underlying cyst formation, suggesting novel therapeutic approaches [5].

This review aims to provide a comprehensive analysis of the epidemiology, pathophysiology, clinical presentation, diagnostic methods, and treatment approaches for arachnoid cysts. We will also discuss current controversies, notable case studies, and future research directions to enhance the understanding and management of these lesions in neurosurgical practice.

## Review

Epidemiology and prevalence

Arachnoid cysts are relatively common intracranial and spinal lesions, with a reported prevalence of approximately 1-2% in the general population. Advances in neuroimaging techniques, particularly MRI, have facilitated the increased detection of these cysts, many of which remain asymptomatic and are discovered incidentally. Despite their benign nature, some cysts can become clinically significant, depending on their size, location, and potential to exert mass effect on adjacent neural structures [6].

Epidemiological studies suggest that arachnoid cysts exhibit a notable male predominance, affecting males approximately two to four times more frequently than females. The majority of cases are congenital, often detected during childhood, although many remain undiagnosed until adulthood when neuroimaging is performed for unrelated symptoms. In pediatric populations, the prevalence ranges from 1.4% to 2.6%, with cysts frequently identified before the age of ten. The prevalence in adults appears to be slightly lower, possibly due to spontaneous regression of some cysts over time or a lack of symptom development that prompts medical evaluation [1,7].

Although most arachnoid cysts occur sporadically, familial clustering has been reported, suggesting a potential genetic component in some cases. Syndromic associations have been documented, particularly with connective tissue disorders such as Marfan syndrome and neurofibromatosis type 1 (NF1), which may predispose individuals to cyst formation. Genetic studies have proposed that mutations affecting extracellular matrix integrity and CSF dynamics could contribute to the development and potential enlargement of these cysts [5].

Most arachnoid cysts remain stable over time, with longitudinal studies reporting stability rates of over 80-90% in both pediatric and adult populations [1,8]. However, approximately 10-20% may enlarge, particularly in younger patients, likely driven by mechanisms such as CSF accumulation, microhemorrhages, osmotic gradients, or a one-way valve effect permitting CSF inflow without outflow [1,8,9]. Enlargement can lead to intracranial hypertension, hydrocephalus, or progressive neurological symptoms, necessitating closer surveillance and potential intervention. Although rare, spontaneous regression has been observed, especially in neonates and young children, possibly due to gradual CSF absorption, cyst rupture, or collapse after minor trauma [9,10]. These patterns emphasize the importance of individualized long-term monitoring based on cyst behavior and patient risk factors.

Anatomy and pathophysiology

Arachnoid cysts arise within the arachnoid membrane, one of the three meningeal layers that surround the brain and spinal cord. The arachnoid mater lies between the dura mater and pia mater and plays a crucial role in CSF circulation. These cysts form when CSF becomes trapped within a duplication or splitting of the arachnoid membrane, creating a fluid-filled cavity that can expand over time. Histologically, the walls of arachnoid cysts are composed of a thin layer of flattened arachnoid cells without an epithelial lining, distinguishing them from other cystic lesions such as epidermoid or dermoid cysts. They do not produce CSF but rather contain fluid closely resembling CSF in composition. Unlike tumors or neoplastic cysts, they lack mitotic activity or proliferative potential, reinforcing their classification as benign, non-neoplastic entities [11].

While their exact etiology remains debated, most arachnoid cysts are considered congenital, arising from developmental anomalies during embryogenesis, whereas a minority are acquired due to trauma, infection, or iatrogenic factors [12]. The prevailing theory regarding congenital arachnoid cyst formation suggests that they develop due to abnormal splitting or duplication of the arachnoid membrane, leading to a CSF-filled space that lacks normal communication with the subarachnoid space. Some authors have proposed a "ball-valve" mechanism, in which CSF enters the cyst through a small defect but cannot exit, causing gradual enlargement over time. Others suggest that osmotic gradients or microhemorrhages contribute to cyst growth [13,14].

Intracranial arachnoid cysts most commonly occur in the middle cranial fossa, particularly the Sylvian fissure, which accounts for approximately 50-60% of cases [15]. Other frequently involved sites include the posterior fossa, suprasellar region, interhemispheric fissure, convexity, and sellar region. Supratentorial cysts, especially those in the Sylvian fissure, are more often diagnosed in children and may exert mass effect on adjacent cortical structures, potentially leading to seizures, developmental delays, or obstructive hydrocephalus [16]. Suprasellar cysts, due to their proximity to the optic chiasm and hypothalamus, can cause visual disturbances, endocrine dysfunction, and CSF flow obstruction [17]. Posterior fossa cysts may compress the cerebellum or brainstem, resulting in ataxia, cranial nerve deficits, or symptomatic hydrocephalus.

Spinal arachnoid cysts are relatively rare, comprising only 1-3% of all cases, and most commonly arise in the thoracic or cervical spine [15,18]. They may be congenital or acquired secondary to trauma, inflammation, or prior surgery. These cysts can compress the spinal cord or nerve roots, leading to myelopathy, radiculopathy, progressive motor or sensory deficits, and gait disturbances [18].

Although many arachnoid cysts remain stable, a subset may enlarge and exert mass effect on adjacent structures, leading to clinical symptoms. Enlargement may occur due to increased CSF production within the cyst, limited absorption, or microtrauma-induced inflammatory changes. In rare cases, cyst rupture or hemorrhage can occur, further complicating clinical management. The relationship between cyst size and symptomatology is not always straightforward, as some large cysts remain asymptomatic, while smaller cysts located in critical regions may cause significant neurological deficits [19].

Understanding the anatomical and pathophysiological mechanisms underlying arachnoid cysts is essential for developing appropriate treatment strategies. As our knowledge of the genetic and molecular mechanisms involved in their formation expands, new therapeutic approaches may emerge, potentially allowing for targeted, minimally invasive treatments that address cyst enlargement and symptom progression without requiring extensive surgical intervention [20].

Clinical presentation

The clinical manifestations of arachnoid cysts vary widely, ranging from asymptomatic cases discovered incidentally to severe neurological deficits requiring urgent intervention. The symptoms largely depend on the size, location, and mass effect exerted by the cyst on adjacent neural structures. While many cysts remain clinically silent, symptomatic cases may present with headaches, seizures, cognitive disturbances, or motor deficits, particularly when the cysts are large or obstruct CSF flow [7,21].

Headache is the most common symptom associated with arachnoid cysts and can occur due to raised intracranial pressure, meningeal irritation, or direct compression of pain-sensitive structures. The headache pattern may be chronic or intermittent, often worsening with postural changes or exertion. In cases where the cyst induces hydrocephalus by obstructing CSF circulation, headaches may be accompanied by nausea, vomiting, and papilledema, necessitating immediate neurosurgical evaluation [22].

Seizures are another frequent presentation, particularly in cases involving supratentorial arachnoid cysts located in the Sylvian fissure, convexity, or interhemispheric fissure. The mechanism by which cysts contribute to epilepsy is not fully understood, but proposed theories include cortical irritation, local neurochemical imbalances, and perilesional gliosis. Studies suggest that between 10-30% of patients with arachnoid cysts present with seizures, and while some respond to antiepileptic medications, others may require surgical intervention, particularly if there is associated cortical dysplasia or structural distortion [22].

Cognitive and behavioral disturbances can occur in both pediatric and adult populations, especially when cysts exert pressure on the frontal or temporal lobes. In children, symptoms may include developmental delay, attention deficits, learning disabilities, and behavioral issues, whereas adults may present with memory impairment, mood changes, or executive dysfunction. There is ongoing debate regarding the direct causative role of arachnoid cysts in cognitive decline, as some studies suggest that the presence of a cyst alone does not always correlate with neurocognitive dysfunction, whereas others report significant improvement following surgical decompression [23,24].

The location of the cyst also influences symptomatology. Posterior fossa cysts can cause balance disturbances, vertigo, dysarthria, and cranial nerve compression, leading to hearing loss, facial weakness, or difficulty swallowing. Large cysts in this region may also impair CSF drainage, resulting in obstructive hydrocephalus and increased intracranial pressure. Though less common, suprasellar and sellar arachnoid cysts can compress the optic chiasm or hypothalamus, leading to visual disturbances, endocrine dysfunction, or precocious puberty in children [25].

Spinal arachnoid cysts are uncommon but can present with radiculopathy, myelopathy, or progressive motor and sensory deficits, depending on the level of spinal cord involvement. Patients may report back pain, weakness, gait disturbances, or bowel and bladder dysfunction. Unlike cranial cysts, spinal arachnoid cysts are more likely to cause symptoms due to the confined space within the spinal canal, making even small cysts clinically significant [26].

Arachnoid cysts may also lead to acute neurological deterioration in rare cases, particularly if they rupture, hemorrhage, or cause a subdural hygroma. These complications are often triggered by trauma, especially in cysts that exert significant mass effect. Spontaneous hemorrhage within the cyst has also been reported and may present with sudden-onset headache, altered consciousness, or focal neurological deficits, necessitating emergency neurosurgical intervention [27-30].

The broad spectrum of clinical presentations underscores the complexity of managing arachnoid cysts. While many cysts remain asymptomatic and do not require intervention, symptomatic cases necessitate individualized treatment strategies, ranging from conservative monitoring to surgical decompression, depending on the severity of symptoms and risk of progression. Continued research is needed to better define the relationship between cyst characteristics and clinical outcomes, aiding in the development of evidence-based guidelines for diagnosis and management [20].

Diagnosis

The diagnosis of arachnoid cysts primarily relies on neuroimaging techniques, as these lesions are often asymptomatic and discovered incidentally during brain or spinal imaging performed for unrelated reasons. When symptoms suggest the presence of an intracranial or spinal lesion, neuroimaging plays a crucial role in differentiating arachnoid cysts from other CSF-containing abnormalities such as porencephalic cysts, neuroglial cysts, enlarged perivascular spaces, or cystic tumors [21].

Neuroimaging Modalities

MRI is the gold standard for diagnosing arachnoid cysts due to its superior soft-tissue contrast and ability to differentiate CSF from surrounding brain structures. Arachnoid cysts appear as well-circumscribed, non-enhancing, CSF-filled lesions that follow CSF signal intensity on both T1- and T2-weighted MRI sequences. Fluid-attenuated inversion recovery (FLAIR) sequences confirm the cystic nature by demonstrating a lack of signal intensity changes compared to surrounding CSF, ruling out proteinaceous or hemorrhagic content [3].

Computed tomography (CT) can also detect arachnoid cysts, particularly when MRI is unavailable or contraindicated. CT scans typically reveal hypodense, non-enhancing lesions with smooth margins, without evidence of calcifications or mass effect unless the cyst is large. However, CT is limited in its ability to differentiate arachnoid cysts from other CSF-containing lesions such as epidermoid cysts or neuroglial cysts, which may require MRI for definitive characterization [3].

Advanced Imaging Techniques

Cine phase-contrast MRI and DWI have emerged as valuable tools for further evaluating arachnoid cysts. Cine phase-contrast MRI assesses CSF flow dynamics, which can help determine whether a cyst communicates with the subarachnoid space. This distinction is crucial in cases where CSF diversion procedures, such as endoscopic fenestration or shunting, are considered [31,32].

Diffusion-weighted imaging (DWI) is valuable for differentiating arachnoid cysts from epidermoid cysts, which can appear similar on conventional MRI sequences. Arachnoid cysts do not restrict diffusion, whereas epidermoid cysts demonstrate high signal intensity on DWI, helping to establish the correct diagnosis [33].

Differential Diagnosis

Several intracranial and spinal lesions may mimic arachnoid cysts on imaging, necessitating a thorough radiological evaluation to establish an accurate diagnosis. Porencephalic cysts, which arise from perinatal brain injury or infarction, can resemble arachnoid cysts but typically have an irregular shape and direct communication with the ventricular system. Neuroglial cysts, though rare, are also CSF-filled but may show focal contrast enhancement or perilesional gliosis. Cystic neoplasms, such as pilocytic astrocytomas or hemangioblastomas, often contain solid components or contrast enhancement, distinguishing them from purely cystic lesions [34].

Spinal arachnoid cysts require differentiation from Tarlov cysts, meningeal diverticula, and intradural cystic tumors. Tarlov cysts, typically found at the sacral level, are perineural cysts that contain CSF but are often asymptomatic. Meningeal diverticula can be congenital or acquired and may fluctuate in size, whereas intradural cystic tumors, such as ependymomas or dermoid cysts, often have solid components or hemorrhagic content, helping to distinguish them from spinal arachnoid cysts [35].

Clinical Correlation and Diagnostic Challenges

Despite the advancements in imaging techniques, correlating radiological findings with clinical symptoms remains a challenge. Some patients with large arachnoid cysts remain asymptomatic, whereas others with small cysts located in critical regions experience significant neurological deficits. Given this variability, careful clinical evaluation, serial imaging, and consideration of cyst size, location, and CSF flow dynamics are essential in guiding management decisions [20].

While neuroimaging is often sufficient for diagnosis, some cases may require lumbar puncture or CSF analysis to rule out alternative etiologies, particularly in patients with suspected infection, neoplasm, or inflammatory conditions. However, lumbar puncture should be performed with caution in cases where there is suspicion of intracranial hypertension, as sudden CSF drainage can exacerbate mass effect or lead to herniation [36].

Overall, early and accurate diagnosis is crucial in determining the appropriate management approach for arachnoid cysts. While most cysts are benign and require only periodic observation, symptomatic cases demand a multimodal assessment that integrates neuroimaging, clinical evaluation, and careful exclusion of alternative diagnoses. As imaging techniques continue to evolve, future research may provide more refined diagnostic criteria and predictive models to improve clinical decision-making and treatment outcomes [21].

Treatment approaches

The management of arachnoid cysts is highly individualized, depending on factors such as symptom severity, cyst size, location, and potential for progression. While many cysts remain asymptomatic and require only observation with periodic imaging, others may necessitate surgical intervention to relieve mass effect, restore CSF flow, or prevent complications such as hydrocephalus and seizures [7,19,37,38].

Conservative Management

For asymptomatic or minimally symptomatic cysts, a conservative approach is often recommended. These patients undergo regular clinical and radiological follow-up, with MRI scans at intervals of 6 to 12 months initially, followed by less frequent monitoring if stability is confirmed. Cysts that show no significant growth or mass effect generally require no intervention, as studies indicate that most remain stable over time with an excellent prognosis [38]. However, conservative management is not suitable for patients with progressive symptoms, hydrocephalus, or focal neurological deficits, as delaying intervention in such cases may result in irreversible damage [20,38].

Surgical Indications

Surgical treatment is considered in patients who develop intracranial hypertension, seizures, cognitive dysfunction, motor deficits, or progressive cyst enlargement. Specific indications are listed in Table 1 [26].

**Table 1:** Surgical Indications for Arachnoid Cysts CSF = Cerebrospinal fluid

Surgical Indications for Arachnoid Cysts
Symptomatic hydrocephalus due to CSF flow obstruction
Seizures refractory to medical treatment with suspected cyst-related cortical irritation
Cognitive or behavioral disturbances associated with cyst-induced mass effect
Cyst rupture, hemorrhage, or subdural hygroma formation
Spinal cord compression or radiculopathy due to spinal arachnoid cysts

Surgical Techniques

Several surgical techniques are available for the treatment of arachnoid cysts, with the choice of procedure depending on cyst location, size, and surgeon expertise. Each approach has distinct advantages and limitations.

Endoscopic fenestration is a minimally invasive procedure that involves creating an opening in the cyst wall, allowing CSF to communicate freely with the subarachnoid space. This approach is preferred for supratentorial and midline cysts, particularly those in the Sylvian fissure, suprasellar region, and posterior fossa. The advantages of endoscopic fenestration include shorter recovery times, reduced surgical morbidity, and lower risk of infection compared to open craniotomy [39]. However, cyst recurrence remains a concern, as fenestration may close over time, requiring reoperation.

Microsurgical fenestration via open craniotomy provides direct access to the cyst, allowing for the creation of multiple drainage windows and, in some cases, partial or complete excision of the cyst membrane. This approach is often used for large or multiloculated cysts that may not be adequately treated with endoscopic techniques. While more invasive, microsurgical fenestration has a lower recurrence rate and allows for better visualization of adjacent neurovascular structures. However, the risks include longer hospital stays, the potential for CSF leaks, and higher surgical morbidity [40].

In cases where fenestration is unsuccessful or contraindicated, cystoperitoneal (CP) shunting may be performed. This technique involves placing a catheter from the cyst to the peritoneal cavity, allowing for continuous CSF drainage. Shunting is typically reserved for large, symptomatic cysts or those causing hydrocephalus, particularly when direct fenestration is not feasible. However, shunts are prone to complications, including infection, overdrainage, mechanical failure, and dependence on lifelong shunt function [41].

For spinal arachnoid cysts, surgical treatment involves laminectomy with cyst excision or fenestration to relieve spinal cord compression. The decision to intervene is based on progressive myelopathy, radiculopathy, or significant mass effect. Complete excision is preferable when feasible, as partial fenestration may not always prevent recurrence [26,42].

Emerging and Experimental Therapies

As neurosurgical techniques advance, novel approaches to treating arachnoid cysts are being explored. Minimally invasive, image-guided procedures are being refined to improve surgical precision while reducing patient morbidity. Additionally, research into gene therapy and molecular targets may provide insight into the pathophysiology of cyst formation and potential pharmacologic interventions [43,44].

Postoperative Outcomes and Complications

The prognosis following surgical intervention is generally favorable, with most patients experiencing significant symptom relief. Studies indicate that 73-82% of patients undergoing fenestration or shunting report improvement in headache, hydrocephalus, and neurological deficits [45,46]. However, potential complications include cyst recurrence (particularly after fenestration), shunt-related complications (e.g., infection, obstruction, and overdrainage), CSF leakage and subdural hygroma formation, surgical site infection and bleeding, and postoperative seizures, especially in cases of cortical irritation [47].

Long-term follow-up is essential to monitor for recurrence, assess symptom progression, and optimize treatment outcomes. Although most patients with successfully treated symptomatic cysts experience stable neurological function, continued surveillance remains important, particularly in pediatric cases where cyst growth is more likely [1].

Conclusion on treatment strategies

Overall, the long-term management of arachnoid cysts requires a highly individualized approach, guided by cyst size, location, clinical presentation, and risk of progression. Most asymptomatic or minimally symptomatic patients can be safely observed with periodic neuroimaging, while those with progressive symptoms, high-risk locations, or significant mass effect warrant early surgical evaluation. Emerging advances in minimally invasive techniques, predictive modeling, and a better understanding of cyst pathophysiology are helping to refine treatment strategies. These developments promise more targeted, risk-stratified management approaches that optimize neurological outcomes while minimizing surgical risk [20,48].

Complications and Risks

While arachnoid cysts are generally benign and slow-growing, they can lead to neurological complications in cases where they expand, exert mass effect, or interfere with CSF dynamics. Additionally, surgical interventions, though often effective, are associated with specific risks and potential long-term consequences. Understanding these complications is critical for optimizing treatment strategies and patient outcomes [19].

Complications of Untreated Arachnoid Cysts

The natural progression of arachnoid cysts is highly variable, with most remaining asymptomatic. However, in some cases, untreated cysts may lead to neurological deterioration due to progressive enlargement, CSF flow obstruction, or the development of secondary pathologies.

One of the most significant complications is hydrocephalus, which occurs when the cyst blocks normal CSF circulation, leading to increased intracranial pressure. Patients may experience headaches, nausea, vomiting, visual disturbances, and altered consciousness, requiring urgent neurosurgical evaluation. Hydrocephalus is more commonly associated with suprasellar, posterior fossa, and quadrigeminal cistern cysts, which are in proximity to the ventricular system [49].

Seizures are another potential complication, particularly in supratentorial arachnoid cysts that exert pressure on the adjacent cortical structures. Although the exact mechanism remains unclear, cortical irritation, perilesional gliosis, and altered neuronal excitability have been implicated. In some cases, seizures may become refractory to antiepileptic medications, necessitating surgical decompression [50].

Cognitive and behavioral changes, particularly in children with large cysts, have also been reported. Compression of the frontal or temporal lobes can lead to executive dysfunction, memory impairment, attention deficits, and mood disturbances. While the direct causative role of arachnoid cysts in cognitive impairment remains debated, several studies have shown improvement in neurocognitive function following surgical intervention, particularly in pediatric patients [23,51,52].

Spontaneous cyst rupture and hemorrhage, though rare, represent life-threatening complications. Cyst rupture may occur after minor head trauma, leading to acute symptoms due to the sudden release of CSF and intracranial pressure shifts. Hemorrhagic events within the cyst or adjacent subdural space can cause acute headache, focal neurological deficits, and altered consciousness, necessitating emergent surgical intervention [27,29].

Postoperative Complications

Although surgical intervention is often effective in relieving symptoms, it carries inherent risks that must be carefully considered. The most common postoperative complications are described in Table 2.

**Table 2:** Postoperative Complications and Descriptions CSF = Cerebrospinal fluid; CP = Cystoperitoneal

Complication	Description
Cyst Recurrence	Occurs in 10-30% of cases, more likely in multiloculated cysts or impaired CSF resorption, may require repeat surgery [53].
Subdural Hygroma Formation	CSF diversion can cause fluid accumulation in the subdural space, leading to mass effect and symptoms similar to chronic subdural hematomas [54,55].
Shunt-Related Complications	CP shunts may fail due to malfunction, infection, overdrainage, or dependency, potentially causing slit ventricle syndrome or hydrocephalus [55].
Infection and Meningitis	Risk of postoperative bacterial meningitis and wound infections, especially in immunocompromised or pediatric patients, requiring early detection and aggressive antibiotic therapy [55].
CSF Leakage and Pseudomeningocele	Inadequate dural repair may result in persistent CSF leaks, increasing the risk of infection and necessitating further interventions [55-57].
Postoperative Seizures	New-onset epilepsy may develop, particularly in cases involving cortical manipulation, requiring long-term seizure prophylaxis in high-risk patients [58].

Long-Term Prognosis and Management of Complications

The long-term prognosis for patients with arachnoid cysts depends on symptom severity, surgical success, and the presence of postoperative complications. Many patients who undergo surgery experience significant improvement in headache, hydrocephalus, and cognitive function, though outcomes are less predictable in cases involving seizures or behavioral disturbances.

Patients who undergo surgical treatment require long-term follow-up with periodic neuroimaging to assess for recurrence, CSF flow abnormalities, or the development of subdural collections. The timing of follow-up MRI scans varies, but most protocols recommend an initial MRI within 3-6 months postoperatively, followed by annual imaging for 2-5 years if stability is maintained [59].

Non-surgical cases also require clinical and radiological monitoring, particularly in pediatric patients where cysts may enlarge over time. Surveillance is typically performed with serial MRI scans every 1-2 years, with closer monitoring in cases of borderline symptomatic cysts [38].

Conclusion on Risks and Complications

While many arachnoid cysts remain clinically silent and stable, others may lead to serious neurological complications or require surgical intervention. Understanding the potential risks associated with both untreated and surgically managed cysts is essential for optimal patient care. With continued advancements in imaging, minimally invasive neurosurgery, and personalized treatment strategies, the long-term outcomes for patients with symptomatic arachnoid cysts are expected to improve [43].

Case studies and notable research

Over the past several decades, numerous case studies and clinical research findings have contributed to our understanding of arachnoid cysts, particularly regarding their natural history, clinical presentation, surgical outcomes, and potential complications. While large-scale studies provide epidemiological and pathophysiological insights, case reports often highlight unique presentations, rare complications, and novel treatment approaches.

Unique Case Presentations

Several case studies have documented unusual or severe presentations of arachnoid cysts that highlight the diverse nature of these lesions. For example, a case report described a giant left frontal arachnoid cyst with parenchymal compression and gyral deformation in a three-year-old male child, presenting with delays in cognitive advancement and difficulty following commands, naming objects, and making eye contact. MRI confirmed mass effect on the underlying brain structures, and the patient underwent successful left frontal craniotomy for microsurgical fenestration, with marked cognitive and behavioral improvement at one-month follow-up [60].

Another case described a spontaneous rupture of an arachnoid cyst after minor head trauma, leading to the formation of a subdural hygroma with acute neurological deterioration. The patient required urgent craniotomy and subdural drainage, emphasizing the potential for cyst rupture in high-risk cases [29].

In a particularly rare case, an arachnoid cyst in the suprasellar region caused progressive visual impairment due to compression of the left posterior optic nerve and anterior optic chiasm. Endoscopic trans-sphenoidal fenestration of the arachnoid cyst facilitated recovery of her visual acuity to 20/20 in both eyes, as well as substantial improvement of her visual field defects [61].

Recent Clinical Studies on Arachnoid Cyst Management

Several large-scale studies and meta-analyses have assessed the long-term outcomes of different treatment approaches for arachnoid cysts. In 2021, Schulz et al. described surgical management at their institution for intracranial cysts in eight different anatomical regions via a retrospective study from 2007 to 2018. Their findings across 113 pediatric patients supported the use and effectiveness of endoscopic fenestration, especially in deeply located cysts [62].

A 2019 retrospective cohort study of temporal arachnoid cyst in a pediatric population over 25 years yielded 240 cases with 69% of patients electing surgery. Rates of clinical improvement did not significantly differ across approaches (microsurgery, endoscopic cyst fenestration, CP shunting, or subdural shunting). However, the endoscopy group had higher rates of early complications including subdural hematomas, as well as shorter event-free survival [63].

A 2023 retrospective cohort study of 108 patients from 2008 to 2022 found that surgical intervention led to improvement in over 90% of intracranial cases. Improvement was significantly more likely in patients younger than 33.5 years old. Neither microsurgical nor endoscopic approach proved superior to the other. The recurrence rate was 5% [64].

A 2019 meta-analysis on the three most common surgical interventions (microsurgical cyst excision, endoscopic cyst fenestration, and shunting) concluded that all three were effective at reducing or eliminating symptoms, while acknowledging the trade-offs of each approach. For instance, microsurgery is associated with longer hospitalizations and surgery-related trauma due to its invasiveness. Shunting is associated with mechanical shunt-related failures. The endoscopic approach should be favored in arachnoid cysts located in the quadrigeminal cisterns to reduce the risk of damaging the incisural and quadrigeminal veins [65].

Experimental and Emerging Research

Recent advances in molecular biology and neuroimaging have led to new hypotheses regarding the pathogenesis of arachnoid cysts. Research on genetic markers suggests that certain congenital cysts may arise from mutations affecting arachnoid membrane integrity and CSF absorption mechanisms. Studies using single-cell RNA sequencing have identified potential gene candidates involved in CSF compartmentalization, paving the way for future non-surgical therapeutic interventions [44].

Neuroimaging advancements, including high-resolution functional MRI (fMRI) and diffusion tensor imaging (DTI), have improved the ability to assess perilesional brain function. These techniques may help predict which cysts are most likely to cause neurocognitive impairment and guide surgical decision-making in borderline cases [66,67].

Additionally, minimally invasive and robotic-assisted neurosurgical approaches are being explored to enhance the precision and safety of cyst fenestration procedures. These technologies aim to reduce operative morbidity, improve fenestration success rates, and minimize the risk of recurrence [68,69].

Clinical Implications of Research Findings

The growing body of clinical and experimental research continues to refine our understanding of arachnoid cysts. Current evidence supports a conservative approach for most asymptomatic cysts, while surgical intervention remains the mainstay of treatment for symptomatic cases. Advances in imaging, surgical techniques, and molecular research may soon lead to personalized treatment protocols tailored to each patient's specific risk profile.

As more long-term, multicenter studies are conducted, clinicians will have better predictive models to determine which patients require intervention and which can be safely observed. Future innovations, including targeted pharmacologic therapies and regenerative treatments, may eventually eliminate the need for invasive surgery in select cases.

Future directions and unanswered questions

Despite significant advances in the diagnosis and management of arachnoid cysts, several unanswered questions and areas of ongoing research remain. Future studies will focus on refining predictive models for cyst progression, optimizing surgical decision-making, and exploring novel treatment modalities that could reduce the need for invasive interventions.

Natural History and Predictive Models

One of the most pressing challenges in arachnoid cyst management is predicting which cysts will remain stable and which will grow or cause symptoms. Although most cysts do not change over time, a subset (10-20%) may enlarge, leading to neurological deterioration or secondary complications such as hydrocephalus or seizures [1,8]. Currently, no universally accepted criteria exist for determining which cysts will become symptomatic.

Recent studies suggest that artificial intelligence and machine learning models could analyze large-scale imaging and clinical datasets to predict cyst behavior with greater accuracy. These models may incorporate factors such as cyst size, location, CSF flow patterns, and genetic markers to develop individualized risk assessments [44,70].

Improved Surgical Techniques and Alternatives

Surgical intervention remains the mainstay of treatment for symptomatic arachnoid cysts, but current techniques still carry risks such as recurrence, infection, and CSF leakage. Future research aims to improve minimally invasive approaches and develop alternative therapies that may eliminate the need for surgery in certain cases. Examples are listed in Table 3.

**Table 3:** Treatment Strategies Under Investigation and Advantages CSF = Cerebrospinal fluid

Future Treatment Approach	Potential Advantages
Endoscopic-Assisted Robotic Surgery	Enhances precision in fenestration procedures, reduces risk of recurrence and perioperative complications [68,69].
Biodegradable Stents for CSF Flow Regulation	Keeps cyst fenestrations open longer, reducing recurrence risk after surgery [71,72].
Gene Therapy and Pharmacologic Interventions	Targets CSF absorption defects and arachnoid membrane abnormalities to prevent cyst formation without surgery [44].

Neurocognitive and Behavioral Impact

Another critical area of research involves the long-term neurocognitive effects of arachnoid cysts, particularly in pediatric patients. While some studies suggest that surgical decompression can improve cognitive function and behavior, others indicate that cyst presence alone may not always correlate with neurocognitive deficits [23,24]. Ongoing research seeks to clarify how arachnoid cysts in the frontal and temporal lobes affect cognitive development, as well as the long-term neurocognitive outcomes of surgery versus conservative management. Additionally, identifying biomarkers or imaging features that can predict cognitive decline or improvement following intervention remains a key area of investigation. Studies using fMRI and DTI aim to clarify how cysts affect brain connectivity, neuronal plasticity, and regional brain function, providing a better foundation for treatment recommendations [66,67].

Long-Term Follow-Up and Surveillance Protocols

Currently, no standardized follow-up guidelines exist for patients with asymptomatic arachnoid cysts, leading to variations in imaging surveillance protocols. Some studies suggest that patients with stable cysts and no neurological symptoms may require less frequent follow-up imaging, while those with cysts in high-risk locations (e.g., suprasellar or posterior fossa) should undergo regular monitoring [19]. Future research aims to define the optimal follow-up intervals for stable, asymptomatic cysts, evaluate the role of advanced neuroimaging (e.g., phase-contrast MRI) in monitoring CSF flow changes, and determine when early intervention may be warranted based on predictive biomarkers.

Pediatric Versus Adult Management Strategies

Arachnoid cysts behave differently in children compared to adults, with pediatric patients exhibiting higher rates of cyst enlargement and potentially greater cognitive and developmental impact. However, current treatment guidelines do not fully differentiate between pediatric and adult cases. Areas for improvement include defining age-specific surgical indications, establishing neurodevelopmental monitoring strategies, and conducting longitudinal studies tracking pediatric patients into adulthood [1,7].

Unanswered Questions in Pathogenesis

Although arachnoid cysts have been traditionally classified as congenital or acquired, their underlying pathophysiology remains incompletely understood. Emerging research suggests that certain cysts may develop due to subtle genetic abnormalities in arachnoid membrane formation, while others may expand due to inflammatory processes or previous minor trauma. Additionally, CSF flow abnormalities, pressure gradients, or local osmotic effects may contribute to cyst enlargement over time [12]. Further histopathological and molecular studies will be necessary to confirm these mechanisms and determine whether early intervention or preventive treatments could halt cyst progression in high-risk individuals.

The Future of Arachnoid Cyst Research

The future of arachnoid cyst research lies in refining diagnostic and predictive tools, improving minimally invasive treatments, and developing novel therapeutic approaches that minimize the need for surgery. With the advent of machine learning, molecular genetics, and advanced neuroimaging, clinicians may soon be able to predict cyst behavior with greater accuracy, identify patients at risk for progression, and tailor treatment approaches to each individual's needs.

While surgical intervention remains the primary option for symptomatic cysts, future developments in non-surgical therapies, pharmacologic modulation of CSF dynamics, and regenerative medicine may provide alternative treatment strategies, reducing the need for invasive neurosurgical procedures.

As more long-term, multicenter clinical trials are conducted, the neurosurgical community will gain a clearer understanding of which cysts require intervention and which can be safely observed. With continued advancements, the goal is to develop a personalized, evidence-based approach to managing arachnoid cysts, improving both neurological outcomes and quality of life for affected patients.

## Conclusions

Arachnoid cysts are common intracranial and spinal lesions, typically asymptomatic but occasionally causing neurological symptoms such as headaches, seizures, or hydrocephalus. Management decisions are guided by cyst size, location, symptomatology, and risk of progression.

Most cysts can be monitored conservatively, while symptomatic cases may require surgical intervention. Endoscopic fenestration, microsurgical fenestration, and cystoperitoneal shunting are the primary surgical options, with the choice depending on cyst characteristics and surgeon expertise. Prognosis after treatment is generally favorable, though complications such as recurrence or infection may occur. Advances in imaging, artificial intelligence, and molecular research are improving risk stratification and may lead to minimally invasive, targeted therapies. A multidisciplinary approach remains essential to optimize outcomes and tailor management strategies for patients with arachnoid cysts.
